# Supplementary material for: Mortality and Costs of Cardiac Implantable Electronic Device (CIED) Infections According to the Therapeutic Approach: A Single-Center Cohort Study
Source: Microorganisms. 2024 Mar 7;12(3):537. doi: 10.3390/microorganisms12030537 (PMC10974394; doi:10.3390/microorganisms12030537)
Supplement: Supplementary file 1 [file microorganisms-12-00537-s001.zip › microorganisms-2830275-supplementary.pdf]

Supplementary material Patients and Methods:

Data collection

From 1985 to 1998 the cases were recorded from the Infectious Disease Service, most of them being of systemic infections. From the year 1999 onwards, a specific intervention program was implemented for these cases, which was developed by a multidisciplinary team (cardiologists, cardiac surgeons, infectious diseases specialists and anesthetists). Infectious diseases (ID) specialist was contacted when a CIED-related infection was suspected (from revisits, Emergency Departments or Primary Care physicians). After the patient examination, the expert ascertained the type and priority of the infection. Similarly, cases from other centres were evaluated by the multidisciplinary team, agreeing with their referring physician the intervention process. Data were recorded prospectively and systematically, depending on a previously established protocol registered in a database designed for that purpose.

Microbiological procedures

Two batches of serial blood cultures (baseline and after 30 and 60 minutes) were performed for each patient at a 12-hour interval. If local signs were presented, an exudate sample through swabs was collected or, in the case of a lack of evidence of outer suppuration, through needle puncture-aspiration in the generator pocket. With the obtained material, a Gram staining and an aerobic and anaerobic culture were performed. In the event of a suspected anaerobic infection, the sample was incubated during two weeks before establishing the culture as negative. When surgery was completed, the distal 4-5 cm of the extracted leads were cultured after sonication and in case of positivity the resulting microorganism was considered to be responsible for the infection. A part of the tips were stored at -20°C and, if cultures were negative, techniques for genomic identification (PCR RNAr16S) were applied.

Antimicrobial treatment

The antimicrobial treatment was established in accordance with the antibiogram of the causative agent isolated in cultures. In local infections, unless drastic symptoms (fever, pain, abundant purulent exudation) or an aggressive microorganism (*S. aureus* or Gram-negative bacilli) was involved, the treatment was started 48 hours before the extraction, maintaining it orally for one or two weeks after TLE was performed. If complete extraction was not performed, therapy duration was individualized and guided by the ID specialist.

In systemic infections, antimicrobial treatment was always started intravenously prior to device extraction, performing the TLE when control blood cultures were negative (usually after a week). If the extraction was complete, the treatment was maintained for two more weeks, allowing the sequencing to oral treatment after the first week, until completion. In the event of valvular affectations (such as tricuspid endocarditis), antimicrobial therapy was maintained for four weeks.

Surgical procedure

All TLE were performed in the same cardiac surgery operating room by two experienced cardiac surgeons and operating room personnel (two nurses, an anaesthetist and a echocardiographer cardiologist), specially trained in that technique, with a tutored

apprenticeship for 2 years. The procedure was always performed under general anaesthesia with tracheal intubation and invasive blood pressure monitorization through a radial artery, as well as jugular or femoral vein cannulation. Prophylactically, two transitory percutaneous pacemaker plates were placed for cases in which asystole was registered in the monitorization and intracardiac stimulation was not available.

When the blood cultures were positive, an echocardiographic study was performed (from 1999 always in transesophageal mode) by cardiologists specifically trained to identify vegetations in leads and/or valvular structures, with image registration. If a pulmonary embolism was suspected, angio-TAC was performed for its verification

In the event of a deep vein thrombosis, a phlebography prior to the extraction was previously performed to check the permeability of subclavian systems, and possible complications during the extraction process were assessed through a blood pressure continuous monitorization and transesophagic echocardiography (severe tricuspid regurgitation, pericardial effusion, or complete eversion of the right ventricle).

Fixative stylets and plastic (propylene) or metallic dissection sheaths (subclavian) were used from 1985 to 1999. Radiofrequency dissection sheaths were used from 1999 to 2010 (Perfecta™, Cook Medical company, Bloomington, IN, USA) and mechanical auto-rotating sheaths in the following years: Shortie™ and Evolution™ (Cook Medical Co) and Tightrail™ (Philips company, San Diego, CA, USA), complemented in some cases with femoral capture handle or clamps inserted from the jugular. Extractions through open cardiac surgery were also performed by the same surgeons involved in the program.

Reimplantation of the electrostimulation device

Each case was evaluated by the electrophysiologist, considering the relevance of the electrostimulation device reimplantation. This procedure was performed either in the same extraction procedure or later. In the case of systemic infections, the only requirement to perform the reimplantation at the same time was the presence of negative blood cultures. If blood cultures were still positive and a promptly extraction was needed (ie: a severe sepsis), in stimulation-dependent patients a femoral transitory system was placed and, once the cultures were negative, a definite contralateral implant was performed at a later date.

Infection types

Depending on their location, infections were classified as:

- Local infection: when systemic symptoms were not present (fever, shock, embolisms, or remote infectious complications), blood cultures were negative and there were signs in the generator pocket such as pain, swelling, erythema and purulent collection objectified by dehiscence of the wound or needle puncture, or exudation by chronic fistula. The cases of a local or partial generator and/or cable extrusion were always considered infected since they are contaminated by skin flora. The pre-decubitus without skin break were considered local infections if during surgery purulent material was observed in the pocket and/or leads or, otherwise, if the cultures from the extracted leads were positive.

- Systemic infection: when the patient had fever, shock, embolisms, or remote infectious complications (such as spondylitis) and the blood cultures were positive. Cases with negative blood cultures required the presence of vegetations in the leads or right-sided cardiac valvular structures, or positive cultures from the distal end of the extracted leads.

Cases with complicated local infections (local symptoms with positive blood cultures) were considered as systemic infections.

Depending on the time of onset, infections were classified as: a) acute: those appearing before 1 month after device implantation or manipulation; b) delayed: those appearing between 2 and 12 months after implantation or manipulation; and c) late: those appearing after 12 months.

Cost analysis

In each episode, the costs were grouped into three expenditure items:

- The stay cost was determined by the days of total registered days (in one or more centers), assigning the price of the stay/day in either conventional hospitalization or intensive care units. In addition, the costs of the used antimicrobial treatment, which in many cases was extended beyond the stay days, and those derived from outpatient visits due to the process were also included.

- The costs related to the material used in the extraction process were also included in the analysis. However, no other material cost used in the operation room was computed.

- Finally, the costs of the new implanted devices after the extraction of the old device were also included. Since the cost of these devices has varied over time, the average costs for each device in the year 2018 were included in this study.

Suplementary tables and figures

T**able S1.** Microbiology and type of infection (Final classification).

**Microorganism**	**Local**<br><br>**N (%)**	**Systemic**<br><br>**N (%)**	**Total**<br><br>**N (%)**
CoNS[a]	101 (43,3)	47 (32)	148 (38,9)
S. aureus	22 (9,4)	50 (34)	72 (18,9)
GNB[b]	16 (6,9)	10 (6,8)	26 (6,8)
Streptococci	1 (0,4)	7 (4,8)	8 (2,1)
Anaerobes[c]	13 (5,6)	0	13 (3,4)
Others[d]	7 (3)	6 (4,1)	13 (3,5)
Polymicrobial[e]	27 (11,6)	25 (17)	52 (13,7)
Not Known	46 (19,7)	2 (1,4)	48 (12,6)
**TOTAL**	**233**	**147**	**380**

[a]Coagulase-Negative Staphylococci
[b]Gram-negative bacilli
[c]*Cutibacterium acnes*: 12, *Fusobacterium nucleatum*: 1
[d]*Corynebacterium spp*: 5, *E. faecalis*: 5, *H. influenzae*: 1, *Microbacterium* spp: 1, *Candida tropicalis*: 1
[e]CoNS and *S. aureus*: 16, CoNS and GNB: 8, various CoNS: 4, CoNS and others: 10, *S. aureus* and GNB: 4, *S. aureus* and others: 6, various GNB: 1, *Coxiella burnetii* and *Brucella spp*: 1, *E. cloacae* and *Corynebacterium spp*:2

Table S2. Microbiology depending on infection onset.

**Microorganism**	**Acute**<br><br>**N (%)**	**Delayed**<br><br>**N (%)**	**Late**<br><br>**N (%)**	**Total**<br><br>**N (%)**
CoNS	493 (32.3)	52 (45.2)	53 (40.2)	148 (38.9)
S. aureus	34 (25.3)	12 (10.4)	26 (19.7)	72 (18.9)
GNB	16 (12.1)	4 (5.4)	6 (4.6)	26 (6.8)
Streptococci	1 (0.8)	1 (0.9)	6 (4.5)	8 (2.1)
Anaerobes	4 (3.0)	6 (5.2)	3 (2.3)	13 (3.4)
Others	2 (1.5)	2 (1.7)	8 (6.1)	1 13 (3.5)
Polymicrobial	19 (14.3)	19 (16.5)	14 (10.6)	52 (13.7)
Not Known	14 (10.5)	19 (16.5)	15 (11.4)	48 (12.6)
**TOTAL**	**133 (100)**	**115 (100)**	**132 (100)**	**380 (100)**

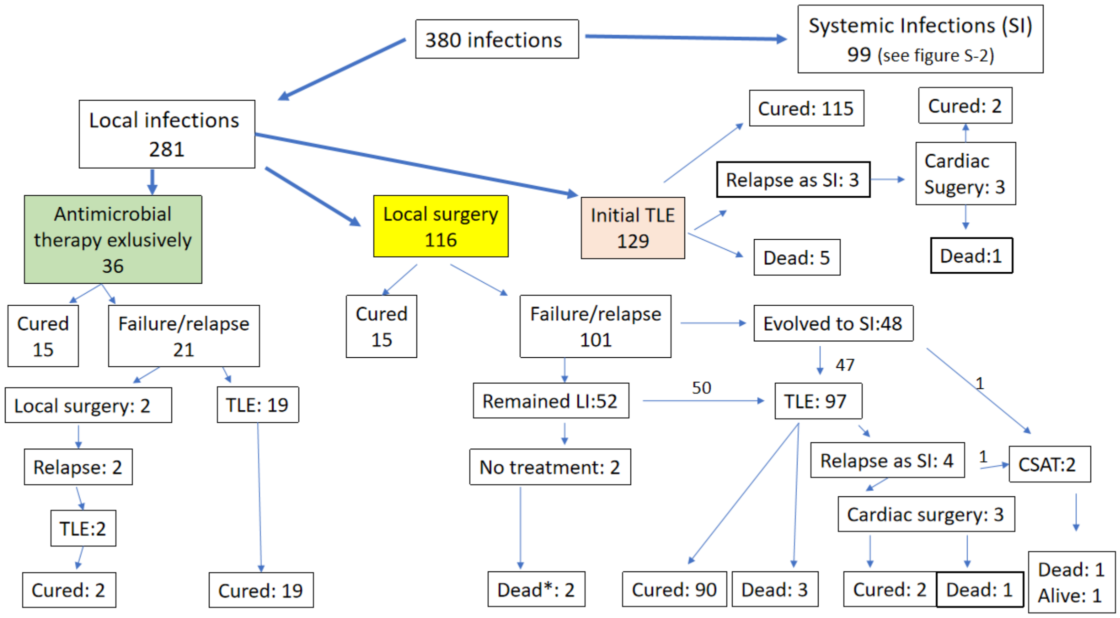

Figure S1: Outcome of the local infections depending of the therapeutic approach (CSAT: Chronic suppressive antimicrobial therapy; TLE: Transvenous Lead Extraction)

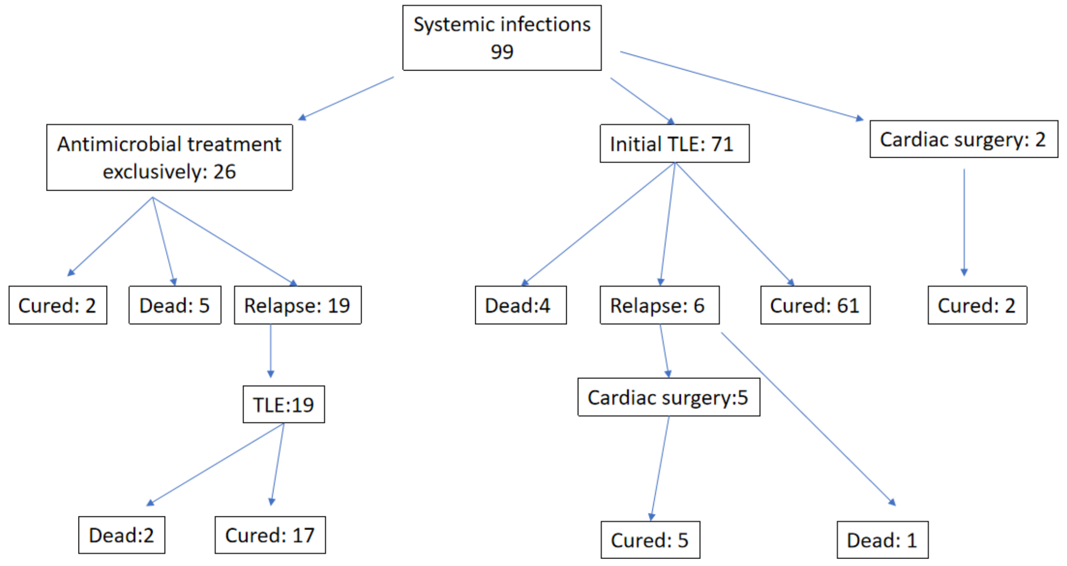

Figure S2: Outcome of the systemic infections depending of the therapeutic approach.

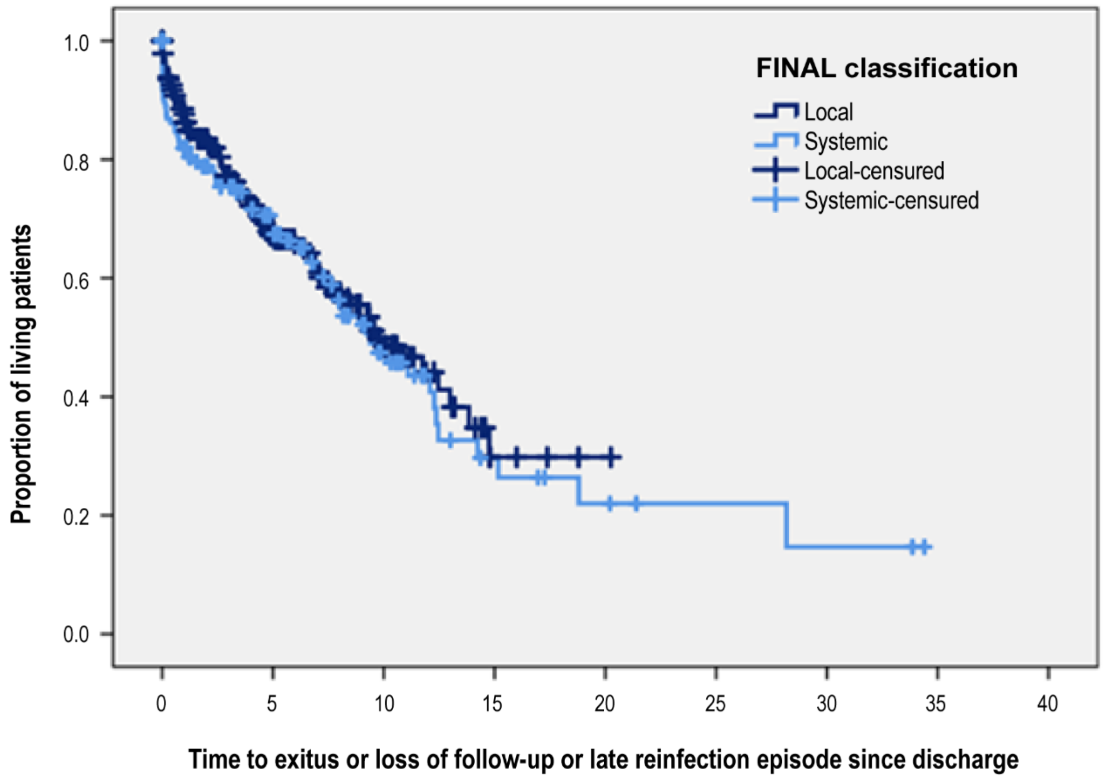

Figure S3. Global survival depending on the type of CIED infection.
